# The influence of contact body size on superficial and deep tissue pain perception for development of a collision dummy

**DOI:** 10.1038/s41598-026-62170-6

**Published:** 2026-07-14

**Authors:** Benjamin Lucas, Roland Behrens, Philipp Echterbeck, Gerald Pliske, Stefan Piatek

**Affiliations:** 1 Department of Trauma Surgery, Otto Von Guericke University Magdeburg, Leipziger Strasse 44, 39120 Magdeburg, Germany; 2 Fraunhofer Institute for Factory Operation and Automation IFF, Sandtorstr. 22, 39106 Magdeburg, Germany

**Keywords:** Algometer, Collision dummy, Pain perception, Topical anesthetic, Anatomy, Engineering, Health care, Medical research, Neuroscience

## Abstract

The use of robots is playing an increasingly important role in industrial manufacturing. Collaborative robots operated alongside humans can pose a hazard. Pain perception as an injury risk indicator is contingent on several factors, especially contact surface, anatomical region and the difference between superficial and deep tissue pain. In this article, we have conducted an experimental load study with human subjects in which we have analyzed these factors to enable the development of a pain-sensitive dummy for testing collaborative robot against biomechanical thresholds. For the load test, we used our established algometer setup with three different contact bodies (7 × 7 mm^2^, 14 × 14 mm^2^ and 24 × 24 mm^2^) to evaluate six anatomical regions of the arm. Subjects were instructed to activate a switch whenever they sensed pain after starting the test procedure. The algometer increased the force in 5 N/s increments. We used topical anesthetic to distinguish between superficial and deep tissue pain. We had 11 male subjects for measurements. The greatest force had to be applied with the medium-sized contact body to induce pain perception. Local anesthetization made it necessary to increase the force of the small and large contact bodies significantly. Our finding that peak force is a reliable indicator of the perception of pain caused by the medium-sized contact body indicates that the contact body’s size affects peak force magnitude significantly.

## Introduction

The use of robots is playing an increasingly important role in industrial manufacturing, especially to cut costs and to improve working conditions. The number of installed industrial robots rose to over four million in 2023^1^. They either work autonomously behind fences or in collaboration with human workers (cobots).

Since they are not separated by fences, cobots can pose a hazard to humans working alongside them. Potential injury severity varies, ranging from pain and hematoma to amputated extremities. In extreme cases, injury can also result in death. ISO/TS 15,066 specifies that a collision between a robot and a person may not exceed their pressure pain threshold (PPT). More severe outcomes, such as injury, are not permissible. Robots’ speed and force consequently must be reduced to within safe biomechanical limits. This necessitates experimentally verified limits that can be applied to protect human workers from injures.

Pain perception as an indicator of injury risk is contingent on several factors. First, nociceptor density plays a crucial role. Nociceptor density affects pressure pain thresholds differently depending on the anatomical region^2^. The literature indicates that body mass index plays a role in perception. Tashani et al. demonstrated that obese individuals have a significantly lower threshold^3^. In addition, the type of stimulus (continuous pressure or brief impact) and the size of the contact surface affect the peak force magnitude that ultimately produces pain. The main nociceptors are the Aδ-fibers and C-fibers. Both fiber types are most abundant in superficial skin, while deeper tissue layers (muscle, bones, etc.) contain mainly C-fibers. The Aδ-fibers are the smallest myelinated nerves and conduct relatively fast a short-lasting and pricking pain. The unmyelinated C-fibers conduct an poor localized and dull pain sensation^4^.

The development of a pain-sensitive collision dummy would be expedient to improve interaction between robots and human workers^5,6^. In our previous articles, we report on our examination of the pain-onset and injury-onset threshold^7–9^. The novelty of this study was to produce experimentally verified PPT for several anatomical regions, which differentiate between superficial pain (from Aδ fibers) and deep pain (from C fibers). The findings can then be used to support the development of a pain-sensitive collision dummy that mimics both fiber layers’ influence on pain perception and supports the development of collaborative robots ensuring a safe interaction and, thus, the deployment of biomechanically safe cobots.

## Material and methods

### Subjects

Subjects were recruited by an announcement in a local newspaper. All applicants were screened based on their medical history and a physical examination, complete blood count and electrocardiogram to rule out blood chaining as well as cardiovascular diseases. Applicants with comorbidities, such as coagulopathy or past injuries, were not accepted as subjects. We only used 11 male subjects for this study to exclude the influence of gender differences on the results as requested by the study funder.

### Experimental setup

**Figure 1:**
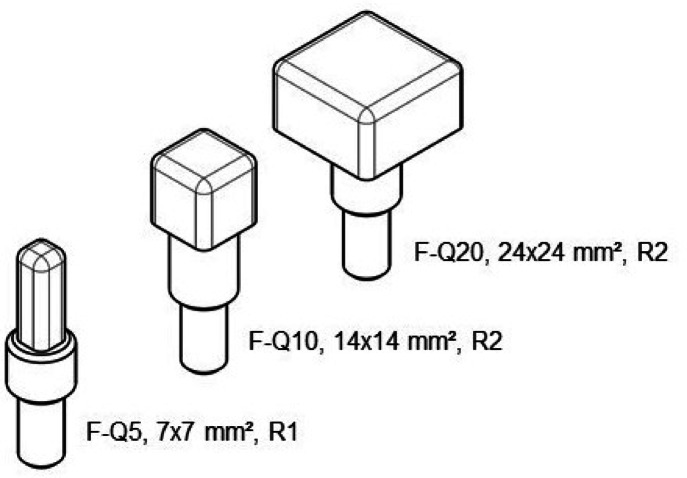
Contact bodies. We used three different contact bodies. The smallest was F-Q5 with an area of 7 × 7 mm^2^, followed by F-Q10 with an area of 14 × 14 mm^2^. The biggest was F-Q20 with an area of 24 × 24 mm^2^.

The general experimental setup and the algometer data processing were similar to that in our group’s last study described in Behrens et al. 2022^8^. In this study, we used three different contact bodies (Fig. 1). All contact bodies were made of aluminum. Contact body F-Q10 was already used in other studies and can be considered a standard in studies that provided thresholds for biomechanically safe cobots^7,8,10,11^. To analyze the effect of the contact area on the PPT, we used two additional contact bodies with the same shape as F-Q10 but different dimensions.

**Figure 2:**
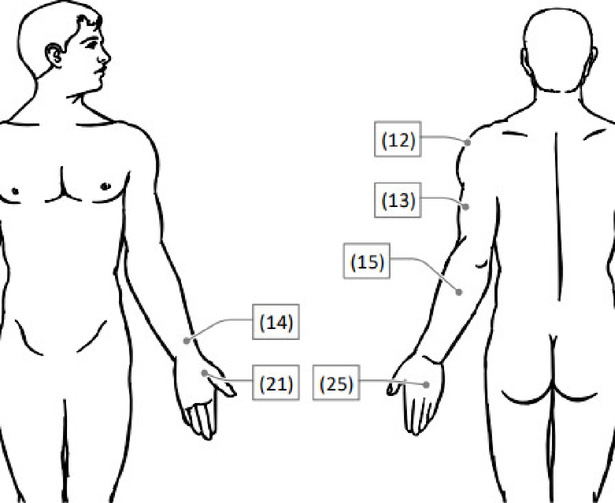
Body locations. Altogether six anatomical regions were tested. Point 12 (deltoid muscle) is located 50–60 mm distally from the acromion in line with the humeral shaft. Point 13 (distal humerus) is located 60 mm proximally from the olecranon on a line from acromion to olecranon. Point 14 (distal radius) is located 40 mm proximally from the foveola radialis on a line leading to a point 10 mm radial from the elbow pit. Point 15 (proximal forearm) is located on the same line 100 mm distally from the elbow. Point 21 (thenar eminence) is located between the radiocarpal joint and the thumb tip. Point 25 (back of the hand) is located between the MCP III and the radiocarpal joint.

Another experimental condition constitutes the body locations tested. Altogether, we evaluated six anatomical regions of the upper arm (Fig. 2). Due to the natural exposure of the arm during manual work, these body locations are most frequently involved in collisions with a cobot.

**Figure 3:**
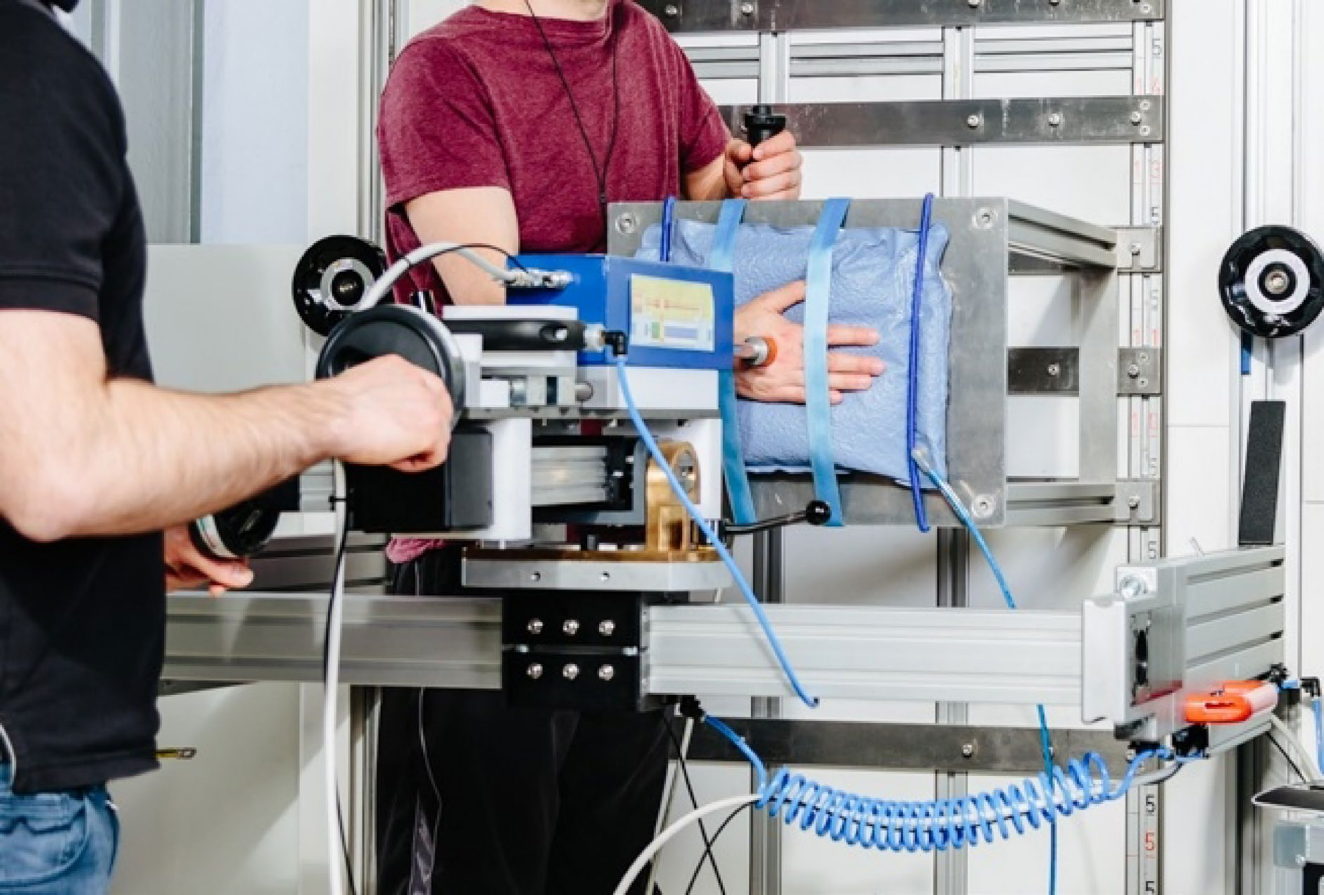
Experimental setup. The algometer was a manually operated device that pressed the contact body against the test subject. Prior to the procedure, the areas to be tested were positioned perpendicular to the algometer’s load axis. The test supervisor then had to increase the force by slowly turning a crank, which pushed the contact body mounted on a rod toward the subject’s body location under test.

The algometer was a manually operated device that pressed the contact body against the test subject. Prior to the procedure, the areas to be tested were positioned perpendicular to the algometer’s load axis. At the beginning of a test, the subjects had to move a two-stage switch to the first position. This activated the force transmission mechanism. The test supervisor then had to increase the force by slowly turning a crank, which pushed the contact body mounted on a rod toward the subject’s body location under test. The force of the algometer was increased with approximately 5 N/s. The subject was instructed to move the pain switch to the second stage once they sensed slight pain in the body location under load whereas the force transmission was immediately terminated and thus the test stopped^8^. Figure 3 shows the experimental setup.

The tests were performed three times with a pause of more than 45 s between each repetition. The test was repeated no later than 24 h later but then the topical anesthetic lidocaine/prilocaine (Emla, Aspen Germany) was applied to the body location.

### Statistics

In the algometer tests, we recorded the contact force measured when the subject moved the pain switch to the second stage. That force was treated as the desired PPT corresponding to level 1 on the numerical rating scale (NRS). All data were processed anonymized in SPSS Statistics (IBM, version 27) and then tested for correlation with a lognormal distribution using the Shapiro–Wilk test. The median and interquartile range of non-normally distributed data and the mean and standard deviation of normally distributed data were box-plotted.

### Ethics

This study was conducted in compliance with the Declaration of Helsinki’s principles. The study protocol was approved by the University of Magdeburg Medical School’s research ethics board (REB#: 37/15 and 13/19). Prior to participating in the study, all subjects gave written informed consent after being briefed on the test procedure. All subjects were insured against injuries that could result from the load tests performed during the study.

## Results

We included 11 male subjects. The epidemiology was presented in Table 1. The subjects’ BMIs were in the normal to preobese range (23.6 ± 1.7 kg/m^2^). Their average age was 34 (± 12.1 years) and mean height was 1.80 ± 0.08 m.

**Table 1:** Epidemiological data of the subjects.

	Number of readings	Range	Minimum	Maximum	Mean	Standard deviation
Age (a)	11	35	23	58	34.09	12.05
Height (m)	11	0.23	1.72	1.95	1.80	0.08
Weight (kg)	11	20.00	66.00	86.00	76.73	6.86
BMI (kg/m^2^)	11	5.24	21.46	26.70	23.57	1.66

### The contact area’s influence on PPT

**Figure 4:**
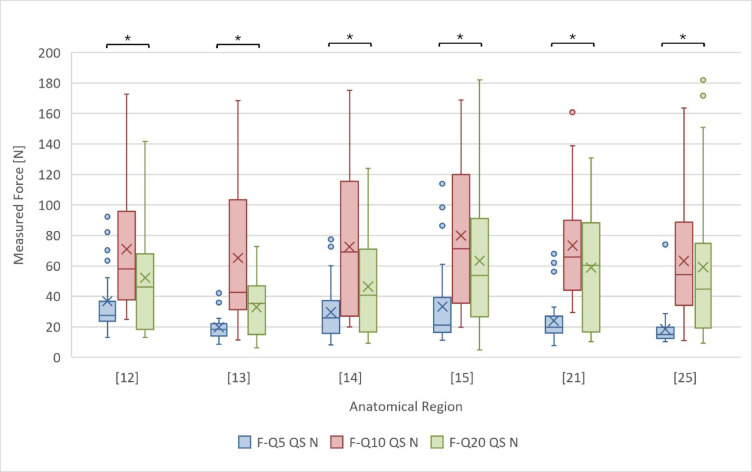
Influence of the contact area on pain perception. Pain perception thresholds in the six anatomical regions without anesthetic was measured for all three contact bodies. The force required for pain to be perceived differed significantly (Friedman test p < 0.001) in all anatomical region depending on the contact body. The medium-sized contact body F-Q10 required the greatest force. line: median, X: mean, box: interquartile range, whiskers: minimum and maximum values. 12—deltoid muscle; 13—distal humerus; 14—distal radius; 15—proximal forearm; 21—thenar eminence; 25—back of the hand.

The force-based PPTs measured in the tests were dependent on the size of the contact body used. Surprisingly, the highest PPTs were observed with the medium-sized contact body F-Q10 in all anatomical regions. The effect of F-Q10 in the distal humerus (13) with a mean of 63.60 ± 45.09 N was stronger than that of F-Q5 with a mean of 19.64 ± 8.28 N or F-Q20 with a mean of 32.22 ± 18.50 N (Friedman test; *p* < 0.001 for any anatomical region; Kendall’s W 0.331–0.579). The detailed results were presented in Fig. 4.

### Differences between superficial and deep tissue pain perception

The PPTs measured at unanesthetized body locations with contact body F-Q5 were significantly lower than the PPTs measured at anesthetized body locations. The greatest difference in PPTs was observed at body location (12),with a mean of 36.09 ± 21.44 N with unanesthetized skin and with a mean of 55.56 ± 40.11 N with anesthetized skin (*p* < 0.001 Wilcoxon-test; r = − 0.749).

**Figure 5:**
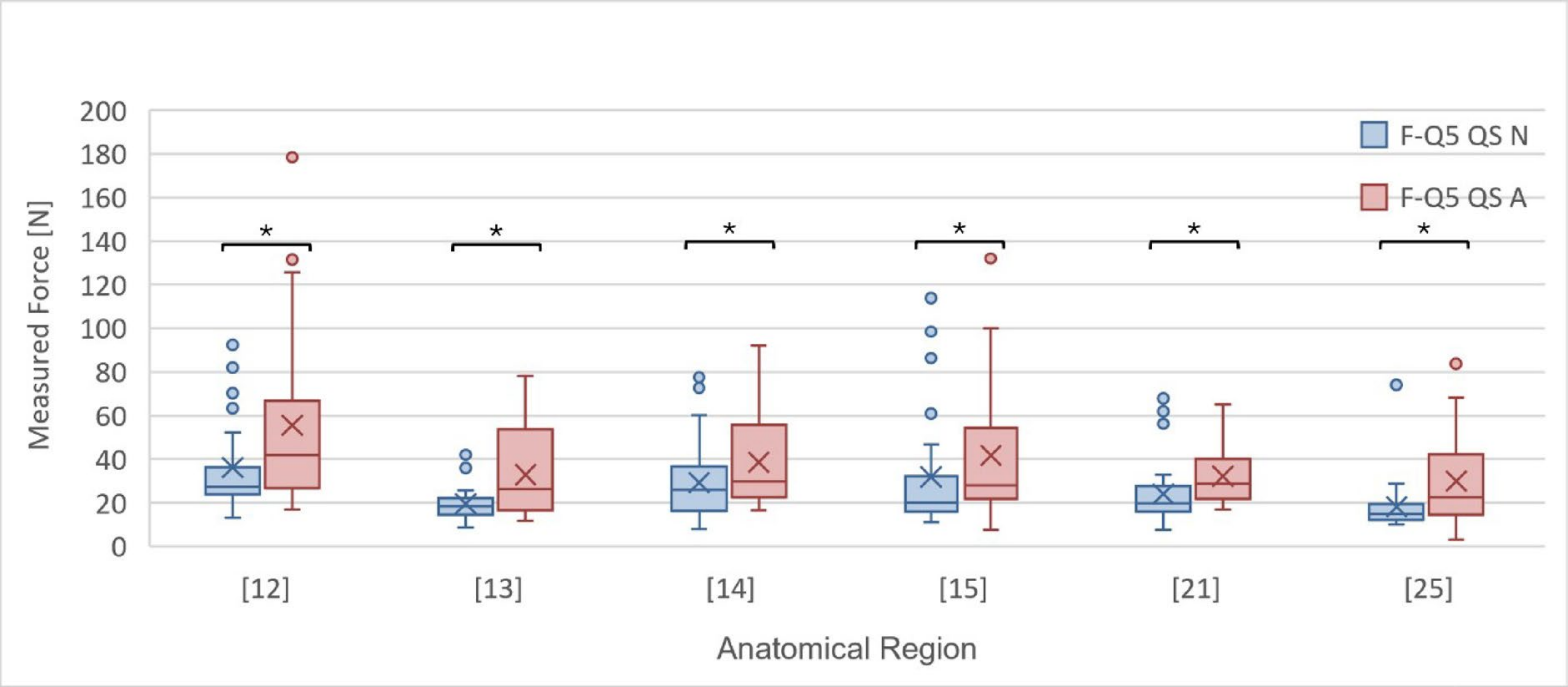
Difference of deep tissue pain perception for F-Q5 contact body. Significantly more force was required from the contact body F-Q5 for pain to be perceived on anesthetized skin (F-Q5 QS A) than on unanesthetized skin (F-Q5 QS N) (p < 0.001 Wilcoxon-test) in all anatomical regions. line: median, X: mean, box: interquartile range, whiskers: minimum and maximum values. 12—deltoid muscle; 13—distal humerus; 14—distal radius; 15—proximal forearm; 21—thenar eminence; 25—back of the hand.

**Figure 6:**
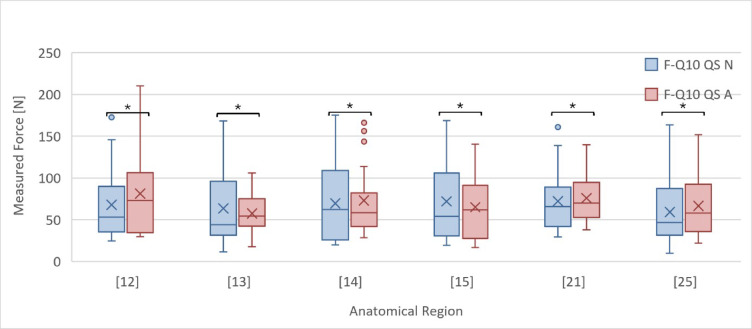
Difference of deep tissue pain perception for F-Q10 contact body. There was no significant difference between anesthetized (F-Q10 QS A) and unanesthetized skin (F-Q10 QS N; *p* > 0.05 Wilcoxon-test) in the anatomical regions tested for the contact body F-Q10. line: median, X: mean, box: interquartile range, whiskers: minimum and maximum values. 12—deltoid muscle; 13—distal humerus; 14—distal radius; 15—proximal forearm; 21—thenar eminence; 25—back of the hand.

**Figure 7:**
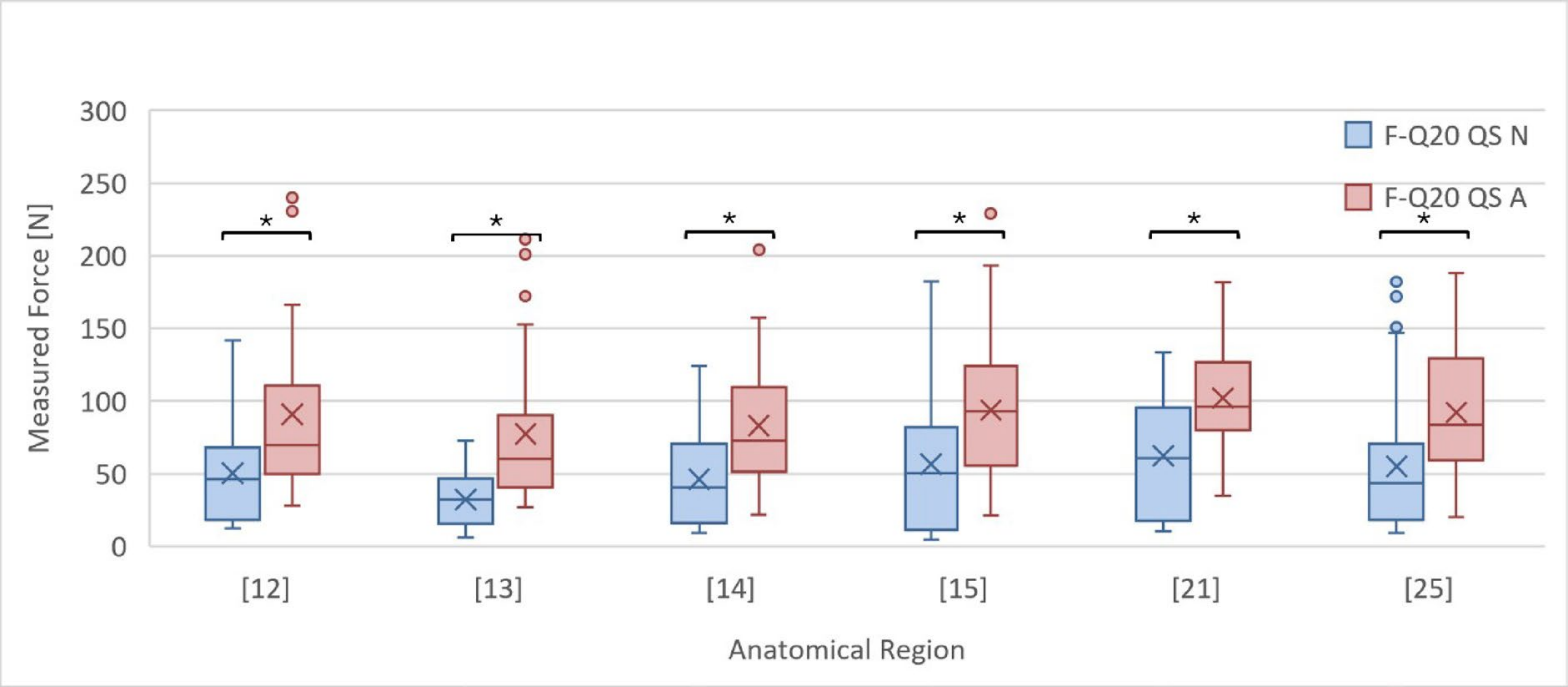
Difference of deep tissue pain perception for F-Q20 contact body. Significantly more force was required from the contact body F-Q5 for pain to be perceived on anesthetized skin (F-Q20 QS A) than on unanesthetized skin (F-Q20 QS N) (*p* < 0.001 Wilcoxon-test) in all anatomical regions. line: median, X: mean, box: interquartile range, whiskers: minimum and maximum values. 12—deltoid muscle; 13—distal humerus; 14—distal radius; 15—proximal forearm; 21—thenar eminence; 25—back of the hand.

There was no clear difference at each measured point for the contact body F-Q10, though. The results for F-Q20 were similar to those for F-Q5. Significantly more force was needed for pain to be perceived on anesthetized skin in all the body regions examined. The PPTs from the test on body location (13) displayed the greatest difference, with a mean of 32.22 ± 18.50 N on unanesthetized skin and with a mean of 77.39 ± 51.07 N on anesthetized skin (*p* < 0.001 Wilcoxon-test; r = − 0.740). The detailed results were presented in Figs. 5, 6 and 7.

To assess intra-subject variability, the three repeated QS measurements obtained under identical combinations of participant, body part, and experimental condition were analyzed. The median within-subject standard deviation was 6.06 N (IQR 2.88–11.27 N), and the median within-subject coefficient of variation was 13.69% (IQR 8.14–20.51%). The median range across the three repeated measurements was 11.48 N (IQR 5.59–21.63 N). Agreement between individual measurements was high [ICC(A,1) = 0.926; 95% CI 0.912–0.938].

## Discussion

In this study, we investigated pain perception thresholds to support the development of a pain-sensitive collision dummy. We specifically investigated the effect of contact area on the PPTs of various body locations under the influence of a topical anesthetic.

The onset of pain serves as a warning signal to the body to prevent physical harm^12^. The direct correlation between the onset of pain and the onset of injury has not been fully explained yet, though^8,13,14^. Behrens and Elkmann 2021 reported that approximately three times more force is required to bring about the onset of injury than to induce slight superficial pain^8,13,14^. Unlike our previous studies^8,14^, in this article we studied PPTs triggered by nociceptive fibers lying in deep tissue by applying a topical anesthetic to the body locations to rule out the influence of nociceptors in the skin.

The literature discusses numerous factors that influence pain perception, such as age^15–18^, gender^18^ or BMI^3^. The usefulness of any pain model of the human body will probably be limited since it is impossible to replicate the entire range of human diversity^19^. Age plays a particularly significant role, older subjects having lower PPTs^15–18^. The subjects tested in this study were aged 23 to 58 years. This age range coincides with that of most of the working population. As shown by Petrini et al.^18^, gender has a significant role when analyzed in combination with age. Younger females tend to have lower PPT than males of the same age. This result could, however, not be confirmed in test with older females and males. Due to the absence of younger female test subjects, the pain-sensitive crash test dummy would be representative only for the target group studied.

Our most recent articles^7,8^ studied thresholds for the onset of blunt injuries and the onset of pain. In this study, we examine the differences between the onset of pain in superficial and deep tissue. Since the nociceptive fibers in skin and deep tissue are different, we conclude, based on our findings, that equipping a pain dummy with at least two sensor layers would be expedient to distinguish between superficial and deep pain when testing cobots against biomechanical limits. Biomechanical thresholds for superficial pain sensation alone would be sufficient since they are always substantially lower to begin with.

In the light of our study’s outcome, we conclude that deep tissue pain perception can be inferred with a topical anesthetic that numbs the superficial pain receptors. The anesthetic produced cutaneous analgesia with a depth of 1 to 2 mm 1 h after application^20^. Our data clearly additionally indicate that the force-based PPT varies with the size of the contact body. For F-Q5 and F-Q20 the PPT was higher on anesthetized skin. However, for F-Q10 we could not observe this difference. Moreover, in case of untreated skin for F-Q10 the PPT was even higher compared to the F-Q20. This finding indicates a transition from superficial to deeper tissue loading. The underlying mechanism has to be investigated in further studies.

## Limitations

During the algometer tests, we ensured that each body location was loaded perpendicularly. Vacuum cushions and straps kept the body part under test from moving. However, physiological differences between subjects could bias the data. Thus, future work should investigate the influence of variation of the contact point position along the arm. The operator was not blinded that could be a source of bias.

The experimenter controlled the increase of the contact force (5 N/s) manually. The procedure was prone to deviation, of course. The contact body’s position was measured over time and the operator tried to maintain a constant increase of the force in all tests. The data from several tests indicate that the experimenter was unable to continuously increase the force manually. This could bias the data because a faster increase would also shorten the subject’s time to react.

As control group we used the untreated skin without anesthesia. A control group with application of control gel applied to the skin was not tested. Another source of measurement error was the subjects’ reaction time when they sensed pain. Even though all subjects were briefed identically on how to react once the contact force exceeded their personal pain-onset threshold, there is no guarantee that they all acted identically. Moreover, pain is subject to several soft factors, such as emotional state and mental health. Before and after each test, each individual had to report their emotional state in a standardized questionnaire. These questionnaires are still being analyzed and the findings will be presented in a separate article focusing on the influence of psychological factors on pain perception.

We investigated only male participants and have to assume that the results were subjected to a gender-specific bias. A Bonferroni correction of the data was not applied to the p values since the *p*-values were all smaller than 0.001.

## Conclusion

The data collected in this study constituted the basis for the development of a pain-sensitive collision dummy. We demonstrated that contact body size significantly influences pain perception. We repeated the tests with anesthetized skin to study deep tissue pain perception. The PPTs from tests with anesthetized skin are significantly higher than the PPTs from tests with unanesthetized skin.

## Data Availability

The datasets generated and/or analyzed in this study are not publicly available because of data privacy regulations but are available from the corresponding author upon request.
